# Genome-wide survey and expression profiles of the AP2/ERF family in castor bean (Ricinus communis L.)

**DOI:** 10.1186/1471-2164-14-785

**Published:** 2013-11-13

**Authors:** Wei Xu, Fei Li, Lizhen Ling, Aizhong Liu

**Affiliations:** Kunming Institute of Botany, Chinese Academy of Sciences, 132 Lanhei Road, Kunming, 650204 China; Graduate University of the Chinese Academy of Sciences, Beijing, China

## Abstract

**Background:**

The AP2/ERF transcription factor, one of the largest gene families in plants, plays a crucial role in the regulation of growth and development, metabolism, and responses to biotic and abiotic stresses. Castor bean (*Ricinus communis* L., Euphobiaceae) is one of most important non-edible oilseed crops and its seed oil is broadly used for industrial applications. The available genome provides a great chance to identify and characterize the global information on AP2/ERF transcription factors in castor bean, which might provide insights in understanding the molecular basis of the AP2/ERF family in castor bean.

**Results:**

A total of 114 AP2/ERF transcription factors were identified based on the genome in castor bean. According to the number of the AP2/ERF domain, the conserved amino acid residues within AP2/ERF domain, the conserved motifs and gene organization in structure, and phylogenetical analysis, the identified 114 AP2/ERF transcription factors were characterized. Global expression profiles among different tissues using high-throughput sequencing of digital gene expression profiles (DGEs) displayed diverse expression patterns that may provide basic information in understanding the function of the AP2/ERF gene family in castor bean.

**Conclusions:**

The current study is the first report on identification and characterization of the AP2/ERF transcription factors based on the genome of castor bean in the family Euphobiaceae. Results obtained from this study provide valuable information in understanding the molecular basis of the AP2/ERF family in castor bean.

**Electronic supplementary material:**

The online version of this article (doi:10.1186/1471-2164-14-785) contains supplementary material, which is available to authorized users.

## Background

The AP2/ERF (APETALA2/ETHYLENE) transcription factor (TF), one of the biggest gene families, contains a typical AP2 DNA-binding domain and exists extensively in plants [1, 2]. The AP2/ERF domain is characterized by approximately 60–70 amino acid residues that constitute a typical helix-turn-helix structure responsible for sequence-specific DNA binding to modulate the target gene expression. Based on the number of AP2/ERF domains and the structural features, the AP2/ERF family is usually divided into four subfamilies (AP2, ERF, DREB and RAV). The AP2 subfamily, containing two repeated AP2/ERF domains, is comprised of two groups, the AP2 group [3] and the AINTEGUMENTA group (ANT) [4, 5]. Their main function involves the regulation of organ-specific growth and development, such as flower development [6], ovule development [4] and the formation of seed size [7], by binding to target sequences gCAC(A/G)N(A/T)TcCC(a/g)ANG(c/t) [8]. Both the ERF and DREB subfamilies contain a single AP2/ERF domain with a specific WLG motif [9]. The ERF subfamily can recognize the conserved nucleotide consensus sequence AGCCGCC of the GCC-box [10] in the promoter regions of pathogenesis-related (PR) genes and modulate their expression in disease resistance signaling pathways [11], whereas the DREB subfamily typically binds to the *cis*-acting elements by the binding sequence CCGAC and is involved in gene expression responsive to abiotic stresses (drought, low-temperature and high salinity) and plant hormones such as ethylene and ABA [12, 13]. The RAV subfamily, containing a single AP2/ERF domain and a specific B3 motif [14–16], is involved in regulating gene expression in response to ethylene [17], Brassinosteroid [18], and biotic and abiotic stresses [19, 20]. In addition, other members containing a single AP2/ERF domain and lacking additional motifs are often named as Soloist. Little is known about their function. Though the identification of structural characterization and the expression profiles for AP2/ERF transcription factors has been extensively studied and documented in several plants such as *Arabidopsis*[9], poplar [21], grapevine [22], a holistic profile of the AP2/ERF family detailing its structure and function in a given species is limited.

Based on genomic sequences, the AP2/ERF family has been characterized in *Arabidopsis*[9], poplar [21], grapevine [22], rice [23], wheat [24] and peach [25]. Castor bean (*Ricinus communis* L. Euphobiaceae) is one of most important non-edible oilseed crops and its seed oil is broadly used in industry. In particular, the main composition of its seed oil is ricinoleic acid, which is considered an ideal and unique feedstock for biodiesel production [26–28]. Due to the increased demand for production of castor bean seed oils in many countries, breeding and improvement of varieties are drawing great attention from breeders [29]. Further efforts should be made to elucidate the molecular mechanism underlying the regulation of growth and development. The recent completion of the castor bean genome [30] provides an opportunity to identify and characterize the holistic profile of the AP2/ERF family, which could add insights into understanding the molecular mechanism of the AP2/ERF family that underlies the regulation of growth and development in castor bean.

A genome-wide survey and characterization of the AP2/ERF family was conducted based on the complete genomic sequences of castor bean in this study. The expression profiles of the AP2/ERF transcription factors among different tissues were examined using high-throughput sequencing for Digital Gene Expression Tag Profiling (DGE). Results obtained from this study provide global information in understanding the molecular basis of the AP2/ERF family in castor bean and other plants in the family Euphobiaceae as well.

## Results

### Detection of AP2/ERF transcription factors in castor bean

In total, 114 putative AP2/ERF transcription factors were identified (see Additional file 1) in castor bean, ranging from 257 to 4877 bp in length. The proteins encoded varied from 85 to 729 aa. According to the number of AP2/ERF domains and their structural features, the 114 proteins can be divided into four subfamilies with 56 members in the ERF subfamily, 34 members in the DREB subfamily, 19 members in the AP2 subfamily, and four members in the RAV subfamily. Like other plants such as *Arabidopsis*, rice, grapevine and poplar, the ERF and DREB subfamilies are the most dominant in castor bean. According to the classification criteria in *Arabidopsis*[9], the DREB subfamily and ERF subfamily can be further classified into six groups each. Within the DREB subfamily the six groups (A1-6) have 6, 5, 1, 10, 7 and 5 members, respectively. Within the ERF subfamily the six groups (B1-6) contain 11, 4, 19, 6, 5 and 11 members, respectively (see Table 1).

**Table 1:** **Summary of the AP2/ERF gene family in** ***Arabidopsis*** **, rice, poplar, grapevine and castor bean**

Plant	Group	Arabidopsis	Rice	Poplar	Grapevine	Castor bean
DREB subfamily	A1	6	10	6	7	6
	A2	8	4	18	4	5
	A3	16	1	2	0	1
	A4	16	15	26	13	10
	A5	16	13	14	7	7
	A6	10	9	11	5	5
	Total	57	52	77	36	34
ERF subfamily	B1	15	16	19	7	11
	B2	5	16	6	3	4
	B3	18	18	35	37	19
	B4	7	9	7	4	6
	B5	8	6	8	4	5
	B6	12	14	16	18	11
	Total	65	79	91	73	56
AP2 subfamily		18	26	26	18	19
RAV subfamily		6	7	5	4	4
Soloist		1	0	1	1	1
Total		147	164	200	132	114

Note: The names of subfamily and group were previously reported by Sakuma et al. in 2002 (see reference [9]). The number shows the number of members in each subfamily and group.

Compared with *Arabidopsis* (147 members), rice (164 members), grapevine (132 members) and poplar (200 members), the AP2/ERF family seems to have relatively fewer members in castor bean. It is obvious that the number of the AP2/ERF members within different subfamilies and groups are varied among species (Table 1). For instance, the number of members in the DREB subfamily ranges from 34 (in castor bean) to 77 (in poplar), and the number of ERF members ranges from 56 (in castor bean) to 91 (in poplar). In addition, one member (30217.m000254) was identified as Soloist, encoded by a single-copy gene with low similarity to the *Arabidopsis* Soloist AT4G13040.

### Conserved residues in the AP2/ERF domain

**Figure 1:**
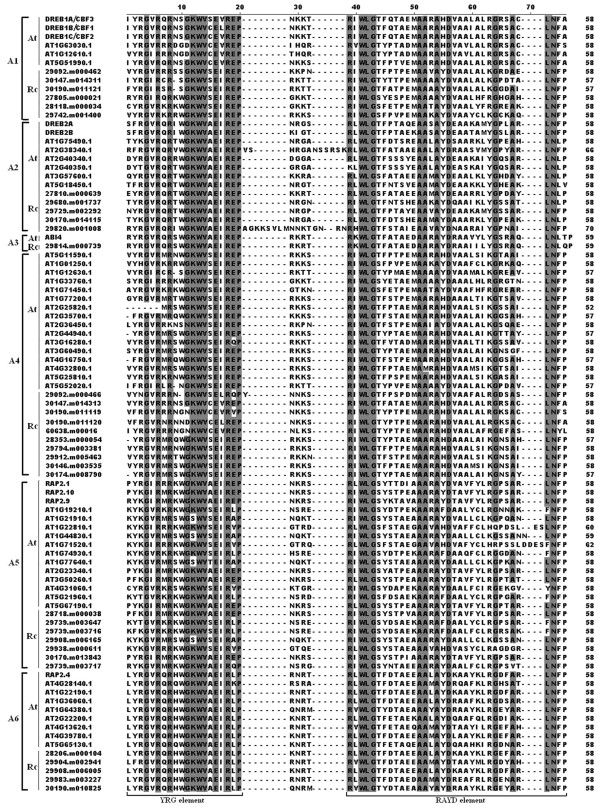
**Comparison of amino acid sequences of the AP2/ERF domains of the DREB subfamily proteins from** ***Arabidopsis thaliana*** **and** ***Ricinus communis*** **.** The black background represents the conserved amino acid residues (>95%). The location of the conserved YRG and RAYD elements are indicated by brackets.

The featured sequences of specific domain regions within transcription factors usually determined the main function of a given transcription factor. Compared amino acid sequences of the AP2/ERF transcription factors from castor bean with *Arabidopsis*, the conserved amino acid residues within AP2/ERF domain were identified for each subfamily. Based on multiple sequence alignments 23 conserved amino acid residues were identified in the DREB subfamily, including 4G, 6R, 8R, 11G, 12 K, 13 W, 14 V, 16E, 18R, 19E, 20P, 39R, 41 W, 42 L, 43G, 51A, 52A, 54A, 56D, 64G, 67A, 73 L, 74 N (see Figure 1). Twenty-three conserved amino acid residues (4G, 5 V, 6R, 11G, 14A, 16E, 17I, 19D, 32 W, 33 L, 34G, 35 T, 42A, 43A, 46Y, 47D, 49A, 50A, 55G, 59A, 62 N, 63 F) were identified in the ERF subfamily (see Additional file 2). In the AP2 subfamily, the sequences of two AP2/ERF domains AP2/ERF-R1 and AP2/ERF-R2 were highly variable because of a 10-aa insertion in the R1 domain or a 1-aa insertion in the R2 domain. However, the linker sequences between the two domains were highly conserved in the AP2 subfamily (see Additional file 3). In the RAV subfamily, both the AP2/ERF domain in the N-terminal region and the B3 domain in C-terminal region were highly conserved (see Additional file 4). In addition, two Soloist proteins identified in castor bean and *Arabidopsis* were highly conserved within the AP2 domain (see Additional file 5). In particular, most of members (more than 90%) in castor bean AP2/ERF family possessed the two featured conserved elements YRG and RAYD elements within the AP2/ERF domain region.

### Phylogenetic and conserved motif analyses

**Figure 2:**
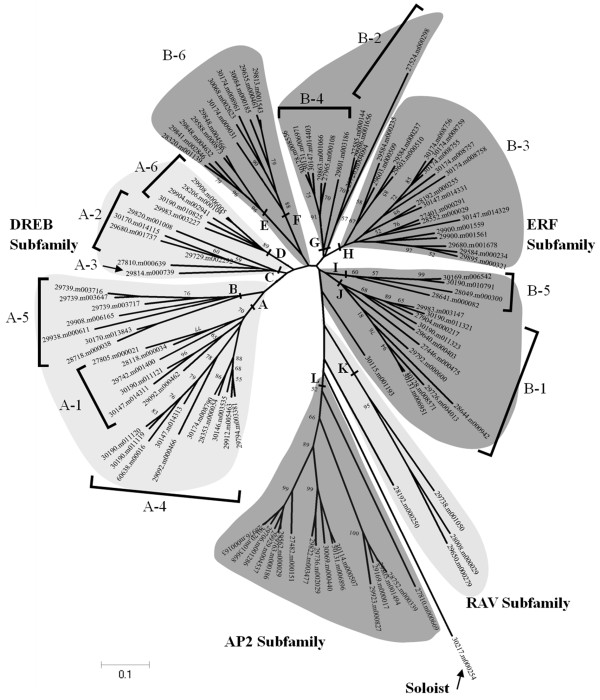
**An unrooted Phylogenetic tree of the AP2/ERF gene family in castor bean.** The amino acid sequences were aligned using Clustal W and the phylogenetic tree was constructed with neighbor-joining criteria. The names of groups (A1-6, B1-6) that have been reported previously are indicated. The letters (A-L) represented the main clades.

To examine the phylogenetic relationships among 114 AP2/ERF members identified in castor bean, an unrooted tree was constructed using MEGA 5.0 with the Neighbor-Joining criteria based on the alignments of full-length protein sequences. The generated phylogenetic tree formed 12 distinct clades (designated A to L) with well supported bootstrap values (Figure 2). It was clearly observed that four clades A-D composed the DREB subfamily, six clades E-J constituted the ERF subfamily, the clade L formed the AP2 subfamily, the clade K made up the RAV subfamily, whereas the Soloist was separated. Within the DREB subfamily, the six groups A1-6 categorized were able to be substantially identified. Similarly, the six groups B1-6 categorized within the ERF subfamily were also substantially identified (see Figure 2).

To dissect the evolutionary relationships of AP2/ERF transcription factors between castor bean and *Arabidopsis*, another unrooted phylogenetic tree was constructed based on the amino acid sequence similarity of 112 AP2/ERF family members in castor bean (excluding 30006.m000282 and 30170.m013669 due to their low similarity) and 120 AP2/ERF family members obtained from *Arabidopsis* in previous study [24]. The phylogenetic tree generated four major clades (designated I to IV, see Additional file 6). Clade I was composed of the AP2 and RAV members; Clade II covered all DREB members (except for the clade II-1clustered by B6 members); Clade III, Clade IV and subclade II-1 included all ERF members; and the Soloist was clustered in Clade II. Further, it was also observed that subclades clustered by groups A1-A6 members of castor bean and *Arabidopsis* within the DREB subfamily and by groups B1-B5 members of castor bean and *Arabidopsis* within the ERF subfamily were substantially identified, but the group B6 members were split in subclade II-1 and other subclades within Clade III. In particular, all major clades and subclades were clustered by interspecies members, indicating that the AP2/ERF transcription factors are homologous between castor bean and *Arabidopsis*.

**Figure 3:**
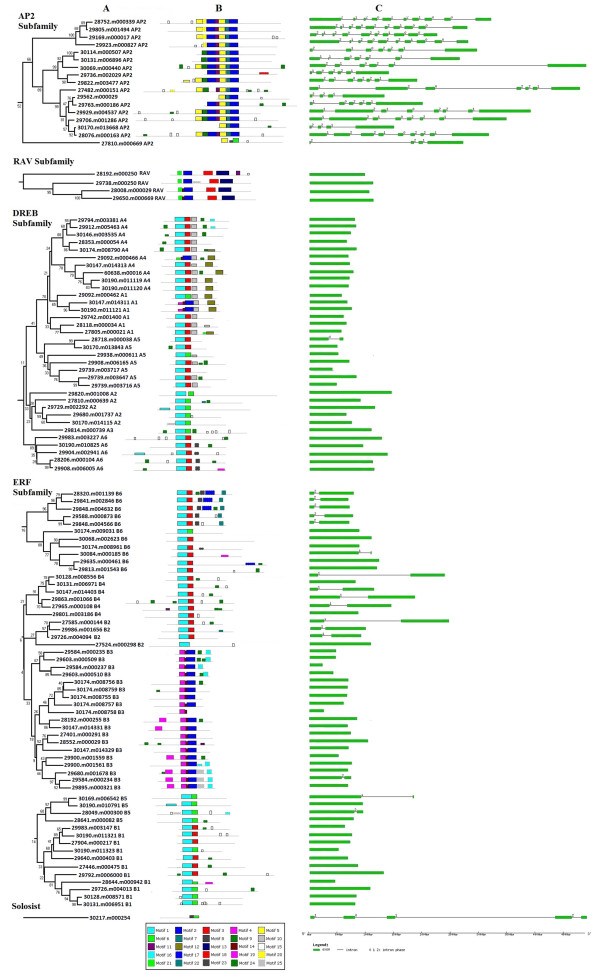
**The structural features and the distribution of conserved motifs within each AP2/ERF subfamily in castor bean. (A)** Phylogenetic clades identified within each AP2/ERF subfamily. **(B)** The distribution of conserved motifs within amino acid sequences of each AP2/ERF gene. The relative positions of each conserved domain within each protein are shown in color. **(C)** Exon/intron structures of castor bean AP2/ERF genes. The exons, represented by green boxes, are drawn to scale. Black lines connecting two exons represent introns. The number above line represents the splicing phases.

The amino acid sequences of AP2/ERF transcription factors contained many conserved motifs which may indicate potential DNA-binding sites or participate in activating the specific function of AP2/ERF genes. Diverse conserved motifs had been identified in *Arabidopsis* and rice [21, 31]. To characterize potential conserved motifs embedded in the AP2/ERF family of castor bean, the 114 complete AP2/ERF amino acid sequences of castor bean were analyzed using the MEME suite version 4.9. In total, 25 conserved motifs were detected and named as motifs 1–25 (see Additional file 7). It was observed that motifs 1–8, 14, 20 and 21 corresponded to the AP2/ERF domain region, and the remaining 14 motifs corresponded to outside of the AP2/ERF domain region. Further, most of the 14 conserved motifs outside the AP2/ERF domain region were nested in specific clades in phylogenetic tree. For example, the motif 9 (characterized in Additional file 8A) was shared by ten members and the motif 11 (characterized in Additional file 8A) was shared by 15 members in the AP2 subfamily; motifs 13 and 18 in the C-terminal region (characterized in Additional file 8B) were specific to the RAV subfamily, and shared by each members in the RAV subfamily; the motif 10 (characterized in Additional file 8C) was shared by A1, A4 and most of A5 members in the DREB subfamily, the motif 23 was specifically distributed within the A6 group in the DREB subfamily; the motifs 16 (characterized in Additional file 8D) and 19 were shared by most of B3 members, and motifs 17 and 22 were shared by several B6 members in the ERF subfamily. These observations indicated that most of conserved motifs were clade-specifically distributed or the members clustered together shared one or more conserved motifs, implying the distinct subfamilies or groups within phylogenetical placement may help in correlating their functions. The distribution of these conserved motifs within proteins of relevant clades in the phylograms was laid out in Figure 3.

### Structural analysis of AP2/ERF genes

Structural analyses of genes revealed that all members in the AP2 subfamily genes had diverse introns ranging from three to nine, whereas 40 of 56 members in the ERF subfamily, 33 of 34 members in the DREB subfamily and all the four members in the RAV subfamily were intronless. Only one was exceptional with a single intron in the DREB subfamily. The 16 members in the ERF subfamily contained just one intron whereas the Solosist gene contained four introns. Further we inspected the pattern of intron positions for those genes containing introns. We found that the positions occurring introns were conserved in both the AP2/ERF domain and the outside AP2/ERF domain regions in the AP2 subfamily, though the number of intron was varied. Similarly, most of genes shared same or similar intron patterns in the ERF subfamily with most introns occurring in the AP2/ERF domain regions (see Figure 3C). The pattern of exon/intron splicing phase usually provided useful information in understanding of the emergence and evolution of gene family. We checked the pattern of exon/intron splicing phase for each intron in the AP2/ERF family. The splicing phases were designated as three splicing phases: phase 0, splicing occurred after the third nucleotide of the codon; phase 1, splicing occurred after the first nucleotide of the codon; and phase 2, splicing occurred after the second nucleotide. Results showed that most members in the AP2 subfamily shared same or similar pattern of exon/intron splicing phase, and the pattern of exon/intron splicing phase also was conserved in the ERF subfamily (see Figure 3C).

Gene structure analyses could provide additional evidence to support the phylogenetic groupings in a given gene family. Our results provided strong evidence to validate our previous phylogenetic groupings. For instance, the five genes (28320.m001139, 29841.m002846, 29848.m004632, 29588.m000873 and 29848.m004566) categorized in the ERF-B6 subgroup shared the same gene structure including patterns of intron position and exon/intron splicing phase (see Figure 3C); the four genes (30174.m008755, 30174.m008756, 30174.m008757 and 30174.m008759) clustered in ERF-B3 subgroup were nearly identical in gene length and structure.

**Figure 4:**
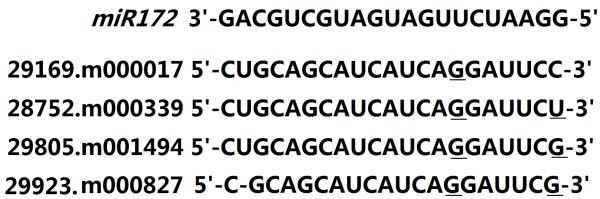
**Putative** ***miR172*** **target sites in mRNAs of AP2 subfamily genes.** The underlined nucleotides denote that it is not complementary to *miR172*.

Recent researches have demonstrated that several AP2 transcription factors are regulated by the microRNA *miR172* in *Arabidopsis*[6, 32]. To reveal potential mechanisms underlying the AP2 subfamily gene regulation in castor bean, we inspected the binding sites targeted by the microRNA *Rc*-*miR172* identified in our previous study [33] for each transcription factor in the AP2 subfamily. The targeted binding sites were unambiguously identified from four genes (29169.m000017, 28752.m000339, 29805.m001494 and 29923.m000827; see Figure 4).

### Expression profiles of the AP2/ERF gene family

To investigate the expression levels of AP2/ERF genes in different organs, high throughput Tag-seq analysis was performed using five tissues leaf, root, seed 1, seed 2 and endosperm (see Methods). The raw sequence data of the five tag libraries obtained from Illumina Genome Analyzer were submitted to the Sequence Read Archive (SRA) under accession SRX343933. In total, 4,574,301, 4,660,289, 4,543,329, 4,650,533 and 4,828,665 clean sequence tags for leaf, root, seed 1, seed 2 and endosperm libraries were obtained (see Additional file 9A). To estimate our sequence quality and sequencing depth, the tag coverage and saturation was analyzed for each library (see Additional file 9). As showed in Additional file 9B, when the sequencing counts reached 2 million tags, the number of detected genes tended towards saturation, meaning that our sequencing depth was sufficient to detect the expression of AP2/ERF genes in each library.

**Figure 5:**
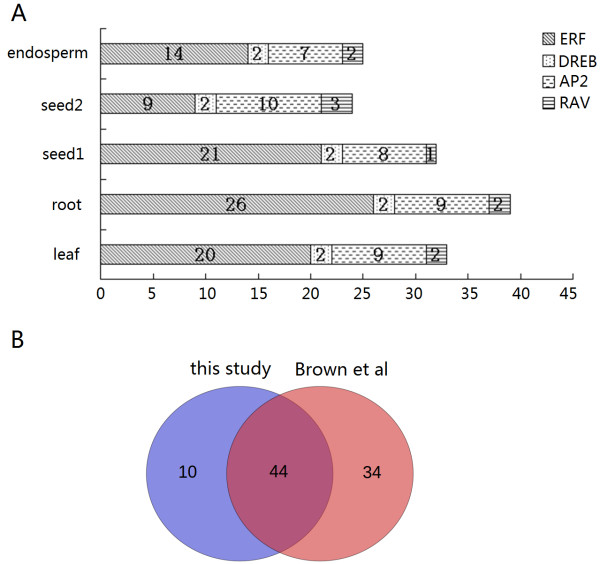
**Number of detected castor bean AP2/ERF genes in leaf, root, seed1, seed2 and endosperm in this study (A) and Overlap of AP2/ERF genes identified in this study and in Brown** ***et al*** **.**[34]**(B)**.

After mapping these clean tags to the castor bean genome database, abundance of tags matching to each AP2/ERF gene regions in five libraries was 1786, 2009, 3046, 569 and 575, respectively. Expression of only 54 AP2/ERF genes was detected in at least one of five tissues tested, covering 32 members in the ERF subfamily, three members in the DREB subfamily, 16 members in the AP2 subfamily and three members in the RAV subfamily. Of them, the expression of 39 genes was detected in root tissue, 33 genes in leaf tissue, 32 genes in seed 1 tissue, 23 genes in seed 2 tissue and 24 genes in endosperm tissue were detected (see Figure 5A). Further, we expanded the expression analysis of AP2/ERF genes using the gene expression database SRA (ERA047687) submitted by Brown et al [34]. As a result, expression of 78 AP2/ERF genes was detected from Brown et al.’s libraries. Compared with our data, the expression of 34 of 78 genes was not detected in our libraries (see Figure 5B). Most of the 34 genes newly detected from Brown et al.’s libraries exhibited a very low expression (see Additional file 10). Combined the two databases, the expression of 88 genes was, in total, detected.

**Figure 6:**
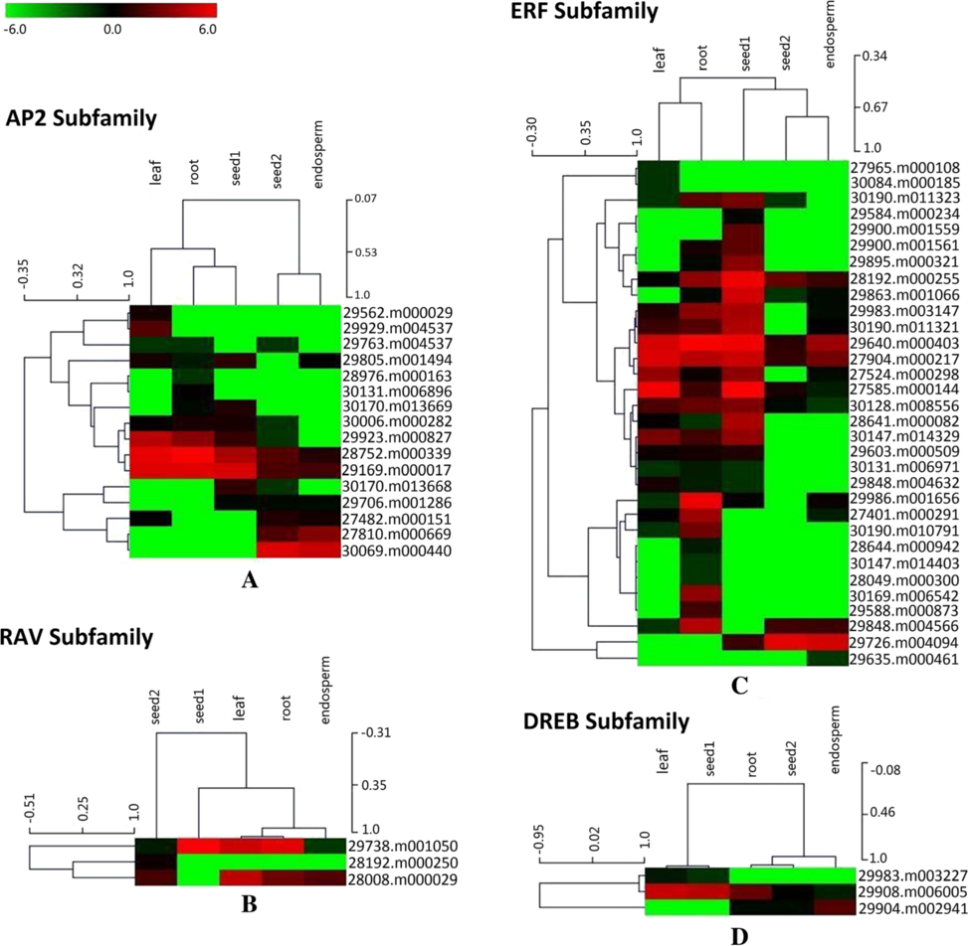
**Heatmaps representing the expression profiles of castor bean AP2/ERF genes in leaf, root, seed1, seed2 and endosperm.** The **A, B, C** and **D** indicate the expression patterns of AP2, RAV, ERF and DREB subfamily, respectively. The log2 signal values of AP2/ERF protein-encoding genes in various tissues/organs and developmental stages (mentioned at the top of each lane) are presented by cluster display. The color scale (representing log2 signal values) is shown at the top.

To understand the temporal and spatial transcription patterns of AP2/ERF genes among different tissues, a hierarchy cluster in each subfamily was separately performed to visualize a global transcription profile of these genes detected from our five libraries. As illustrated in Figure 6, only a few genes (such as 28752.m000339 and 29169.m000017 within the AP2 subfamily, and 29640.m000403 and 27904.m000217 within the ERF subfamily) showed a high expression across five tissues tested. Most of genes exhibited diverse expression profiles among different organs. Further, we combined our transcription data with Brown et al.’s data [34] to profile the expression patterns of AP2/ERF genes in castor bean. Similarly, most of AP2/ERF genes exhibited various expression profiles among different organs. Notably, most of genes exhibited a tissue/organ-specific expression, such as five genes (28320.m001139, 30146.m003535, 28353.m000054, 27810.m000639 and 30174.m008790) specifically expressed in leaf tissue, five genes (28644.m000942, 29562.m000029, 29588.m000873, 29929.m004537 and 30169.m006542) specifically expressed in root tissue, three genes (30131.m006896, 29841.m002846, and 30174.m009031) specifically expressed in flower, four genes (30069.m000440, 30170.m013668, 29726.m004094, and 28192.m000250) specifically expressed in developing seeds, and three gene (29635.m000461, 29680.m001737 and 29736.m002029) specifically expressed in endosperm. Also, 16 genes, which exhibited higher expression levels in vegetative tissues (including leaf and root) than reproductive tissues (including flower, developing seed and endosperm), were identified (see Additional file 10).

Further, we compared the expression profiles of AP2/ERF genes among different organs between castor bean and *Arabidopsis*. Although some AP2/ERF genes and their orthologs (such as 29983.m003227/AT2G20880.1, 28976.m000163/AT4G37750.1, 29584.m000234/AT3G23240.1, 28049.m000300/AT3G61630.1, 29635.m000461/AT5G19790.1, 30084.m000185/AT5G19790.1, 29680.m001737/AT5G18450.1) displayed different expression patterns among tissues, most of AP2/ERF genes and their orthologs presented similar expression profiles among organs between castor bean and *Arabidopsis* (see Additional file 10). Eight genes and their orthologs (29908.m006005/AT1G78080.1, 28752.m000339/AT2G28550.3, 29169.m000017/AT4G36920.1, 27904.m000217/AT3G15210.1, 29640.m000403/AT1G50640.1, 27585.m000144/AT1G53910.1, 28192.m000255/AT4G17500.1, 29738.m001050/AT1G25560.1), for instance, were highly expressed in all organs tested in both castor bean and *Arabidopsis*. In particular, most of genes and their orthologs exhibited a tissue/organ-specific expression profile. For instance, 30069.m000440/AT3G54320.1, 30170.m013668/AT4G37750.1 and 29726.m004094/AT1G72360.1, were preferentially expressed in developing seeds and 29841.m002846/AT1G15360.1 was specifically expressed in flower in both castor bean and *Arabidopsis* (see Additional file 10).

To identify potential transcription factors involved in regulating lipid biosynthesis in developing seeds of castor bean, we purposely analyzed the expressional differences of all transcription factors identified in castor bean (PlantTFDB: http://plntfdb.bio.uni-potsdam.de/v3.0/) between seed 1 (at the initial stage) and seed 2 (at the fast oil accumulation stage) libraries. As shown in Additional file 11, 23 transcription factors significantly up-regulated at the fast oil accumulation stage were identified. In particular, some key regulators of fatty acid biosynthesis, such as LEC1, LEC2, ABI3 and WRINKLED1 were significantly up-regulated, consistent with Brown et al.’s observation [34]. For AP2/ERF genes, 18 genes were significantly down-regulated, and only two genes (30069.m000440 and 29726.m004094) were significantly up-regulated at the fast oil accumulation stage (p < 0.001 and fold-change > 2) (see Additional file 12).

## Discussion

Although the AP2/ERF family has been broadly studied in diverse plants, the current study is the first report on identification and characterization of the AP2/ERF transcription factors based on the genome in the family Euphobiaceae, one important group of resource plants. In total, 114 putative AP2/ERF family genes were identified based on the genome sequences of castor bean. Genome analyses showed that castor bean had undergone recent duplication events [30], which might contribute to the expansion of the AP2/ERF family in castor bean. Compared with *Arabidopsis* (genome size 125 Mb), rice (genome size 466 Mb), grapevine (genome size 490 Mb) and poplar (genome size 480 Mb), castor bean (genome size 310 Mb) harbored the minimum members in the AP2/ERF family (see Table 1). As mentioned above, the AP2/ERF family was extensively involved in regulating plant response to diverse biotic and abiotic stresses. Castor bean can easily grow in diverse habitats from template, subtropical to tropical areas. It appears that castor bean displays a strong tolerance or resistance to diverse environmental stresses. However, why castor bean harbors less members in the AP2/ERF family is yet unknown. The 114 members identified were unambiguously divided into four subfamilies, in consistence with the category of AP2/ERF family in other plants. In particular, Both ERF and DREB are dominant subfamilies containing a single AP2/ERF domain in structure, whereas both AP2 and RAV subfamilies were of minority exhibiting a more complex gene structure such as two AP2/ERF domains and more introns or a specific B3 motif in gene sequences. Probably, an early addition of introns or a second DNA binding domain in structure may have impaired the duplicative ability of the hypothesized ancestral HNH endonuclease in the early evolution of this family, or a longer piece of DNA might have made a transposition and duplication event less likely, resulting in the smaller number of members in the AP2 and RAV subfamilies [35]. In addition, similar to other plants [24, 31], the AP2/ERF domain regions contained many highly conserved amino acid residues in castor bean.

In general, transcription factors functionally result from some important conserved motifs within and outside the DNA binding domain which are related to transcriptional activity, nuclear localization, and protein-protein interactions [31]. Two conserved amino acid residues 14 V/A and 19E/D within the AP2/ERF domain have been proved to be critical for DNA-binding specificity [9]. The identified divergence of amino acid residues 14 V and 19E in the DREB subfamily, or 14A and 19D in the ERF subfamily may be one of the important factors in the understanding of the functional divergence between the ERF and DREB subfamilies in castor bean. In particular, the two elements YRG and RAYD within the AP2/ERF domain had been reported to be critical in activating DNA binding to modulate the expression of target genes in *Arabidopsis*[3, 36]. The two elements YRG and RAYD were highly conserved and identified in most of the members of AP2/ERF family in castor bean, implying their structural and functional necessity. Outside the AP2/ERF domain regions 14 conserved motifs were identified in castor bean AP2/ERF family in this study. Most of these conserved motifs display a group-specific distribution pattern. Combining the structural differentiation of genes among subfamilies or groups, these observations strongly imply that the functional divergence exists among subfamilies or groups. The conserved motifs 13 and 18 may play important roles as transcriptional repressor in mediating plant growth and development [18]. Both motifs 13 and 18 were the RAV subfamily specific, and shared by each member, implying that motifs 13 and 18 may be indispensable elements in structure of RAV subfamily in castor bean. Studies have showed that motifs 9 and 11 could form a long linker of the two β-sheets and these extruded residues or of AP2/ERF proteins and several linker residues in ANT lineage in *Arabidopsis*, which may participate in activating the function of transcription factors in the AP2 subfamily [37]. Both motifs 9 and 11 were the AP2 subfamily specific in castor bean, shared by 10 and 15 members respectively, meaning that motifs 9 and 11 may provide a specific function for DNA binding in the AP2 subfamily in castor bean. The motif 10 was shared by 17 members from groups A1, A4 and A5 in the DREB subfamily, characterized by four blocks of conserved amino acid residues: LPRP, D[IV]QAA/DIR[RA], LRAA and [IHEYQAKS]LNFP (see Additional file 8C). These conserved amino acid residues have been identified to be essential signatures in *Arabidopsis* for CBL-interacting serine/threonine-proteins kinase-12 [38], Ethylene-responsive transcription factor ERF037 [39], dehydration responsive element binding proteins-1C and proteins-G [40], auxin response factor-19 [41], and disease resistance [42], respectively. The motif 16 containing a unique ‘EDLL’ residue was the group B3 specific in ERF subfamily (see Additional file 8D). The ‘EDLL’ residue might participate in activating the function for the group B3 members in the ERF subfamily [43]. However, the function of most conserved motifs identified in castor bean is uncertain. Compared those additional conserved motifs identified outside of the AP2/ERF domain regions in castor bean with other plants, eight motifs (including motifs 9, 10, 11, 13, 16, 17, 18 and 22) were shared by castor bean, *Arabidopsis* and rice, indicating most of additional motifs outside of AP2/ERF domain regions were conserved in plants. The newly identified seven motifs (12, 15, 19, 20, 23, 24 and 25) might be variable among species or species-specific in castor bean.

Phylogenetic analysis of the AP2/ERF transcription factors in castor bean showed that the four subfamilies, AP2, RAV, DREB, ERF, and the main groups A1-A6 within the DREB subfamily, and groups B1-B6 within the ERF subfamily were able to be substantially identified. Compared with the phylogenetical relationships of the AP2/ERF members in *Arabidopsis* and rice [21, 31], phylograms displayed similar clades to our results. The phylogenetical tree generated by the combined members between castor bean and *Arabidopsis* showed a major clade I shared by the AP2 and RAV members, indicating a phylogenetically close relationship between the AP2 and RAV subfamilies. In particular, all major clades and subclades were clustered by interspecies members, indicating that the AP2/ERF transcription factors are homologous between castor bean and *Arabidopsis*. Based on similar gene structure and conserved motifs of AP2/ERF gene in different species, it was indicated that the AP2/ERF transcription factors were highly conserved in angiosperm. These observations strongly support Magnani et al.’s assumption that the AP2/ERF transcription factors might have an ancient origin during angiosperm evolution [35].

As mentioned above, researches have demonstrated that the activities of several AP2 transcription factors are regulated during the development of organs by the microRNA *miR172* in *Arabidopsis*[6, 32, 44]. Our current study identified four AP2 genes containing unambiguous targeted sites for binding *Rc*-*miR172*, which could provide a potential clue to dissect the mechanisms underlying the AP2 gene regulation in castor bean.

Although our sequencing depth was sufficient, high throughput Tag-seq data obtained from five libraries identified the expression of only 54 AP2/ERF genes in this study. Based on the deep RNA-seq data in previous study [34], the expressions of additional 34 AP2/ERF genes were supplemented. The expressional differences of AP2/ERF genes between our data and Brown et al’s data could be explained because of 1) the different sequencing strategy and depth (Brown et al’s data was based on RNA-seq strategy with more deeper sequencing, which was more sensitive for detecting genes expressed at the low level than our Tag-seq strategy [45]); 2) the different tissues tested (Brown et al’s data included flower and geminating seed tissues). The expression profiles of most AP2/ERF genes displayed spatial and temporal expression patterns among different tissues, implying their functional specificity. For example, the 29929.m004537 gene was specifically expressed in root tip tissues, and its homologs (AT5G17430 and AT3G20840) in *Arabidopsis* were functionally involved in regulating the growth and development of root tips [46]. In addition, the expression of 26 of 114 AP2/ERF genes (including 16 members in ERF subfamily, eight members in DREB subfamily, one member in AP2 subfamily, and one member in RAV subfamily) were not detected (see Additional file 10). The possible reasons include: 1) the limited tissues or developmental stages were examined in our analysis, and 2) the tissues tested in both our current examination and Brown et al’s study were sampled from individuals with normal growth (lack of environmental stresses). It is understandable that the expression of some genes in DREB and ERF subfamilies would not be detected if their expressions were majorly involved in responding to biotic and abiotic stresses.

One of main objectives of this study is to identify potential AP2/ERF transcription factors involved in oil accumulation or seed development of castor bean. The analyses of expressional differences between seed 1 and seed 2 libraries revealed that 18 of 20 AP2/ERF genes were down-regulated from the initial developing stage to the oil fast accumulation stage. These genes might be negatively regulated in oil accumulation in the developing seeds of castor bean. For the two genes (29726.m004094 and 30069.m000440) strongly up-regulated at the oil fast accumulation stage, we inspected the function of their the homologs (AT1G72360 and AT3G54320) in *Arabidopsis* and found that AT1G72360 was a hypoxia-inducible ethylene response factor and significantly up-regulated in developing seeds [47–50], and AT3G54320 (WRINKLED1) was a master gene responsible for transcriptionally regulating carbon metabolism and lipid biosynthesis in developing seeds [51–56]. Potentially, the gene 30069.m000440 may be an important transcription factor responsible for regulating oil accumulation in developing seeds of castor bean. Studies focusing on the functional analysis of 30069.m000440 might reveal the mechanism underlying the regulation of oil accumulation in developing seeds of castor bean.

## Conclusions

The current study is the first report on identification and characterization of the AP2/ERF transcription factors based on the castor bean genome in the family Euphobiaceae. In total, 114 putative AP2/ERF family genes were identified in castor bean, one of most important non-edible oilseed crops and its seed oil is broadly used for industrial applications. Further, the 114 AP2/ERF transcription factors were characterized according to the conserved amino acid residues within AP2/ERF domain, the conserved motifs and gene organization in structure, phylogenetical analysis, and global expression profiles among different tissues using high-throughput sequencing. Results obtained from this study provide global information in understanding the molecular basis of the AP2/ERF family in castor bean.

## Methods

### Identification of AP2/ERF transcription factors from castor bean genomic sequences

Based on the castor bean genome (http://castorbean.jcvi.org/index.php), an extensive search was performed to identify all members of the AP2/ERF transcription factors. The *Arabidopsis* AP2/ERF gene and amino acid sequences were downloaded from the DATF database (http://datf.cbi.pku.edu.cn). The characterized ERF sequences from the representative members for each group in *Arabidopsis thaliana*[9] were used as query sequences against the castor bean complete genome using WU-BLAST 2.0 program with an e-value of le-3 and more than 80% coverage. According to the hit position of sequences targeted in castor bean genome, the corresponding gene sequences (including ORF sequences), gene model, position in scaffold, amino acid sequences and their annotations were extracted for further analyses. To obtain an exhaustive search for identifying all members of AP2/ERF family in castor bean, we further used the full length sequences of representative members in other subfamilies, such as AT1G25560.1 representing RAV subfamily, AT2G28550.1 representing AP2 subfamily, and the Soloist AT4G13040 as query sequences for comparing our previous searches. After removing redundant sequences and incomplete ORF sequences, SMART tools (http://smart.embl-heidelberg.de/) and InterProScan (http://www.ebi.ac.uk/Tools/pfa/iprscan/) were used to confirm the presence of the characterized AP2/ERF domain in the candidate sequences. Further, the putative members of AP2/ERF family and their gene sequences were identified and defined for further analyses.

### Phylogenetic, MEME motif and gene structure analyses

Multiple alignments of amino acid sequences of the AP2/ERF domain in *Arabidopsis* and castor bean were carried out using Clustal W [57] and an un-rooted phylogenetic tree was generated with neighbor-joining criteria in MEGA 5.0 [58] with 1000 bootstrap replicates. Conserved motifs in castor bean AP2/ERF transcription factors were identified using motif based sequence analysis tool MEME (Suite version 4.9.0) with the following parameters: optimum width 10–200 amino acids, any number of repetitions of a motif, and maximum number of motifs set at 25. The BLAST search for the resulting motifs in the NCBI and MS-Homology databases was carried out to determine their biological contexts. In addition, gene structure was investigated using the online Gene Structure Display Server (http://gsds.cbi.pku.edu.cn/) based on full-length mRNA alignments with corresponding genomic sequences, whereas introns are gaps between exons consisting entirely of genomic sequence.

### Gene expression analyses

To examine the global expression profiles of 114 AP2/ERF transcription factors identified among different organs or developmental stages, high-throughput sequencing of digital gene expression tag profiles (DGEs) for five tissues leave, root tips, developing seeds at the initial stage (seed 1), developing seeds at the fast oil accumulation stage (seed 2), and endosperm were conducted. Seeds of castor bean var. ZB306 elite inbred line (provided kindly by Zibo Academy of Agricultural Sciences, Shandong, China) were germinated and grown in the conservatory under natural conditions (11 h light, 13 h dark; 25°C during the day and 18°C at night). Mature female flowers were hand pollinated and tagged. Leaf tissues were collected from fully expanded young leaf and root tips were dissected, washed and collected. Immature seeds at two different stages, i.e. seed 1 at the initial stage (developing seeds do not start to accumulate oil, ca. 15 days after pollination) and seed 2 at the fast oil accumulation stage (developing seeds start to fast accumulate oil, ca 35 days after pollination) were collected. Endosperm tissues were dissected from the immature seeds (ca. 40 days after pollination). Three biological replicates were collected for each tissue type. For all the tissues, three randomly chosen samples were pooled to form a biological replicate. Total RNA was isolated from the leaves, roots, seed 1, seed 2 and endosperms of castor bean using Trizol reagent (Invitrogen, Carlsbad, CA) and purified using an RNeasy Mini Kit (Qiagen, Valencia, CA) following the manufacturer’s protocol. The quality of total RNA samples was checked using the NanoDrop Spectrometer (ND-1000 Spectrophotometer, Peqlab) as well as agarose gel electrophoresis.

The high quality RNAs were used to constructed tag libraries respectively for deep-sequencing. Briefly, total ploy A RNA (about 6 μg) was enriched by Oligo(dT) magnetic beads and Oligo(dT) used as the primer to synthesize the first and second-strand cDNA. The cDNA was digested by two types of Endonuclease: NlaIII or DpnII, acquiring 17 bp tags with different adaptors of both ends to form a tag library. After 15 cycles of linear PCR amplification, 105 bp fragments were purified by 6% PAGE Gel electrophoresis. After denaturation, the single-chain molecules were fixed onto the Illumina Sequencing Chip (flowcell). Each molecule then grows into a single-molecule cluster sequencing template through *in situ* amplification. Four colored nucleotides were added for sequencing using the method of sequencing by synthesis (SBS). Millions of raw reads were generated with a sequencing length of 49 bp. Sequencing was performed using a Illumina Genome Analyzer at BGI ShenZhen (China).

The raw data from the five tagged libraries were preprocessed to filter out low quality reads and clipped adapter sequences. After that, all clean reads were mapped to the castor bean genome (http://castorbean.jcvi.org/index.php) to obtain unique reads and reads abundance using SOAP2 software [59]. To compare the differential expression of genes among tissues, the expression level of each gene in the different tissues was normalized to the number of transcripts per million clean (TPM). Genes with significantly different expression were determined by P ≤ 0.001 and fold-change ≥2 in two samples. To visualize a global transcription profile of genes detected in each subfamily across the five tissues, the hierarchical clustering was performed using R software [60].

## Electronic supplementary material

Additional file 1: **Putative 114 AP2 family genes identified in castor bean.** (XLS 54 KB)

Additional file 2: **Comparison of amino acid sequences of the AP2/ERF domains in the ERF subfamily between** ***Arabidopsis thaliana*** **and castor bean.** (DOCX 2 MB)

Additional file 3: **Comparison of amino acid sequences of the AP2/ERF domains in the AP2 subfamily between** ***Arabidopsis thaliana*** **and castor bean.** (DOCX 2 MB)

Additional file 4: **Comparison of amino acid sequences of two domains in the RAV subfamily between** ***Arabidopsis thaliana*** **and castor bean.** (DOCX 566 KB)

Additional file 5: **Comparison of amino acid sequences in the Soloist between** ***Arabidopsis thaliana*** **and castor bean.** (DOCX 170 KB)

Additional file 6: **Phylogenetic relationships of the AP2/ERF genes between Arabidopsis thaliana and castor bean.** (DOCX 1 MB)

Additional file 7: **Conserved motifs identified from the AP2/ERF genes in castor bean.** (DOCX 1 MB)

Additional file 8: **Summary of functionally conserved motifs identified from the AP2/ERF genes in castor bean.** (DOCX 1 MB)

Additional file 9: **Sequencing quality and saturation analysis of the five libraries of root, leaf, seed 1, seed 2 and endosperm.** (DOCX 429 KB)

Additional file 10: **Expressional profiles of AP2/ERF genes among different tissues in castor bean and** ***Arabidopsis*** **.** (XLSX 38 KB)

Additional file 11: **The up-regulated transcription factors identified from seed 1 to seed 2 tissues in castor bean.** (XLSX 14 KB)

Additional file 12: **Expressional difference of AP/ERF genes between the seed 1 and the seed 2 tissues in castor bean.** (DOCX 14 KB)
