# Supplementary material for: Genome-wide survey and expression profiles of the AP2/ERF family in castor bean (Ricinus communis L.)
Source: BMC Genomics. 2013 Nov 13;14(1):785. doi: 10.1186/1471-2164-14-785 (PMC4046667; doi:10.1186/1471-2164-14-785)

**Additional file 2. Comparison of amino acid sequences of the AP2/ERF domains in the ERF subfamily between *Arabidopsis thaliana* and castor bean**


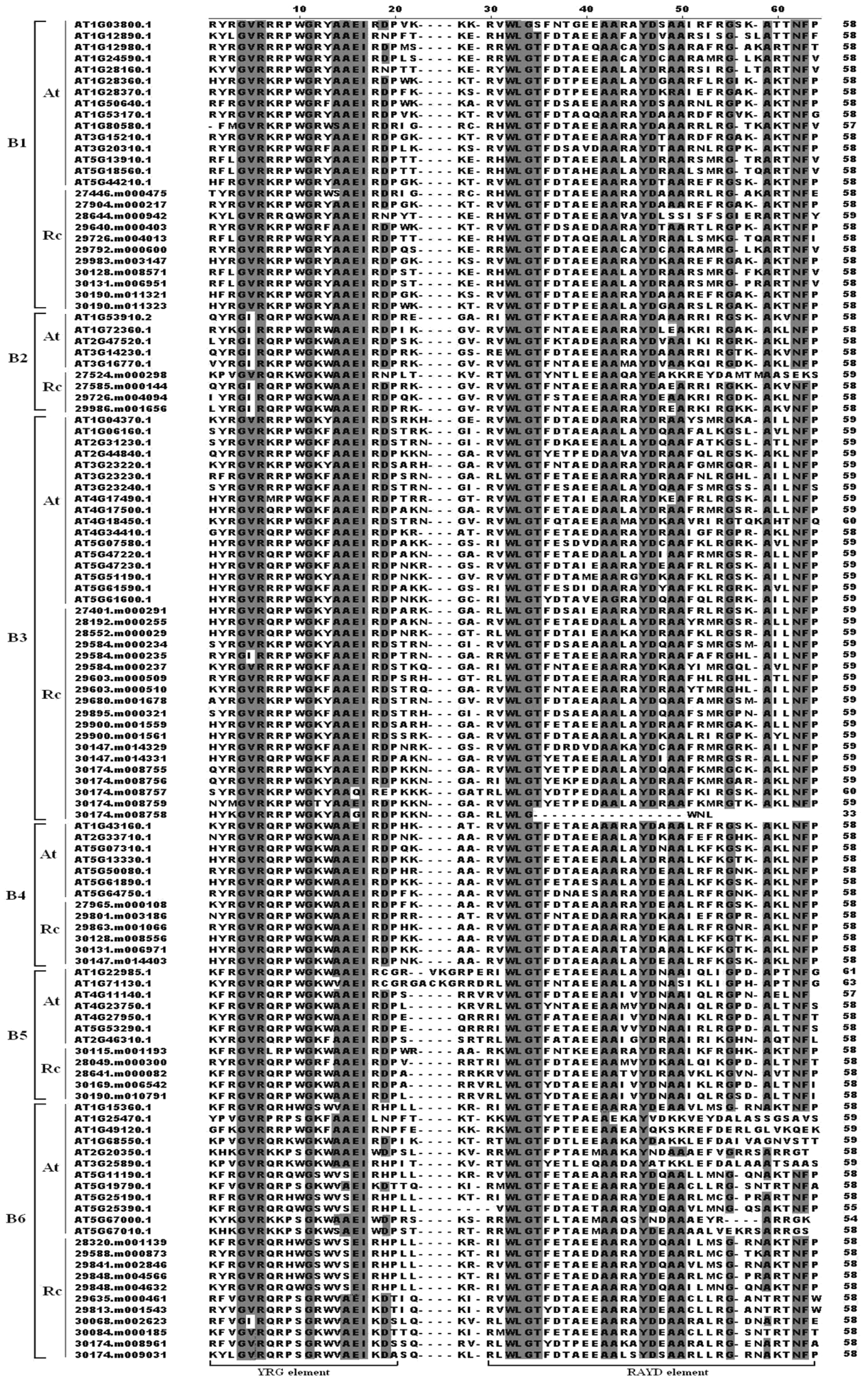

Supplement: Supplementary file 2 — Additional file 2: Comparison of amino acid sequences of the AP2/ERF domains in the ERF subfamily between Arabidopsis thaliana and castor bean. (DOCX 2 MB) [file 12864_2013_5510_MOESM2_ESM.docx]
