# Supplementary material for: Genome-wide survey and expression profiles of the AP2/ERF family in castor bean (Ricinus communis L.)
Source: BMC Genomics. 2013 Nov 13;14(1):785. doi: 10.1186/1471-2164-14-785 (PMC4046667; doi:10.1186/1471-2164-14-785)

**Additional file 4. Comparison of amino acid sequences of two domains in the RAV subfamily between Arabidopsis thaliana and castor bean.**


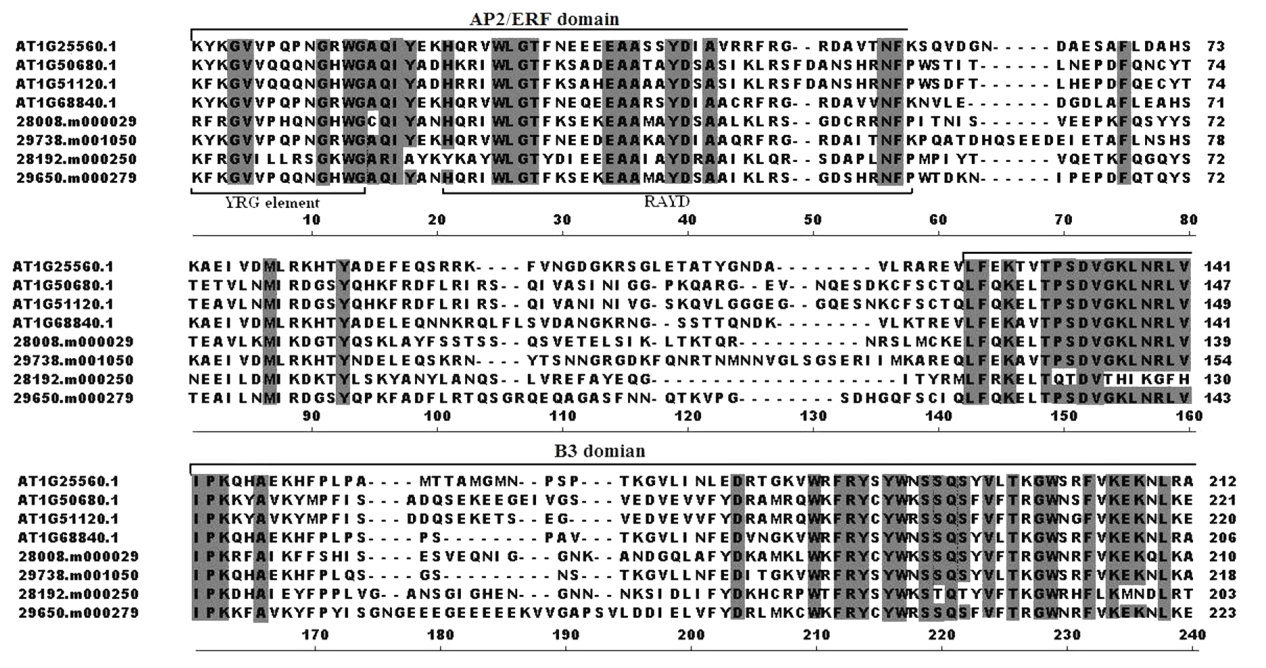

Supplement: Supplementary file 4 — Additional file 4: Comparison of amino acid sequences of two domains in the RAV subfamily between Arabidopsis thaliana and castor bean. (DOCX 566 KB) [file 12864_2013_5510_MOESM4_ESM.docx]
