# Supplementary material for: Genome-wide survey and expression profiles of the AP2/ERF family in castor bean (Ricinus communis L.)
Source: BMC Genomics. 2013 Nov 13;14(1):785. doi: 10.1186/1471-2164-14-785 (PMC4046667; doi:10.1186/1471-2164-14-785)

**Additional file 5. Comparison of amino acid sequences in the Soloist between Arabidopsis thaliana and castor bean.**


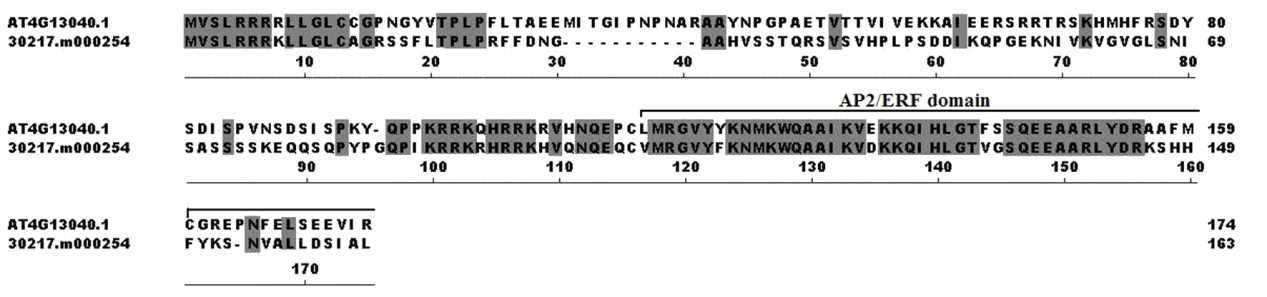

Supplement: Supplementary file 5 — Additional file 5: Comparison of amino acid sequences in the Soloist between Arabidopsis thaliana and castor bean. (DOCX 170 KB) [file 12864_2013_5510_MOESM5_ESM.docx]
