# Supplementary material for: Genome-wide survey and expression profiles of the AP2/ERF family in castor bean (Ricinus communis L.)
Source: BMC Genomics. 2013 Nov 13;14(1):785. doi: 10.1186/1471-2164-14-785 (PMC4046667; doi:10.1186/1471-2164-14-785)
Supplement: Supplementary file 7 — Additional file 7: Conserved motifs identified from the AP2/ERF genes in castor bean. (DOCX 1 MB) [file 12864_2013_5510_MOESM7_ESM.docx]

**Additional file 7. Conserved motifs identified from the AP2/ERF genes in castor bean.**

| MOTIF-1 | 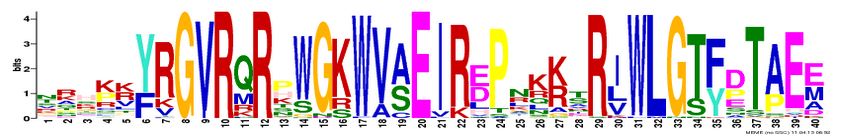 |
| --- | --- |
|  | NRH[KP][KR][YF]RGVRQRPWGKWV[AS]EIR[ED]PNK[KR][TS]R[ILV]WLG[TS][FY][DP]T[AP]E[EM] |
| MOTIF-2 | 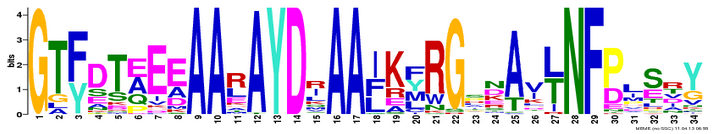 |
|  | GT[FY]DT[EAQ]E[EA]AA[RL]AYD[RI]AA[IFL]K[FYM]RGxNA[VI][LT]NF[PD]L  [SE]RY |
| MOTIF-3 | 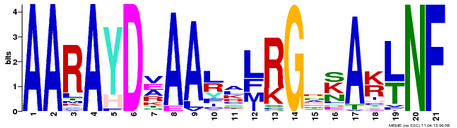 |
|  | AARAYDVAA[LR]x[LF][RK]Gx[KS]A[KR][LT]NF |
| MOTIF-4 | 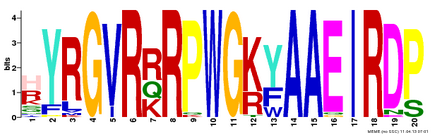 |
|  | [HR]YRGVR[RKQ]RPWG[KR][YF]AAEIRD[PS] |
| MOTIF-5 | 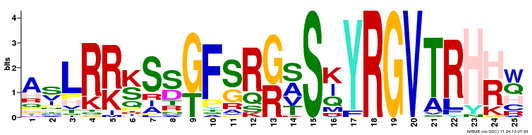 |
|  | ASLR[RK][KS]S[SD][GT]F[SG]R[GR][SA]S[KIQ]YRGVTRH[HR][WQ] |
| MOTIF-6 | 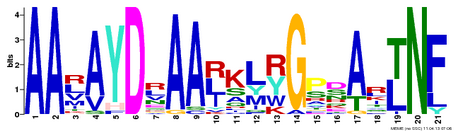 |
|  | AA[LR]AYDRAA[LRI]KL[RY]GPDAR[TL]NF |
| MOTIF-7 | 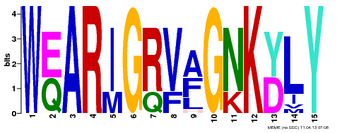 |
|  | W[EQ]AR[IM]G[RQ][VF][AF]G[NK]K[YD]LY |
| MOTIF-8 | 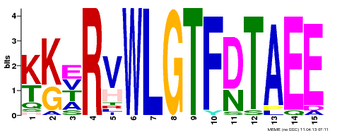 |
|  | [KT][KG][ETV]R[VH]WLGTF[DN]TAEE |
| MOTIF-9 | 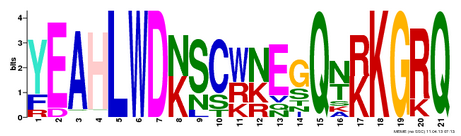 |
|  | [YF]EAHLWD[KN][SN][CS][WRK][NKR]E[GS]Q[NT][KR]KG[RK]Q |
| MOTIF-10 | 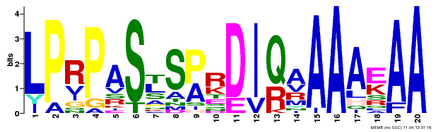 |
|  | LPRPAS[LT]S[PA]RDI[QR]AAAA[EK]AA |
| MOTIF-11 | 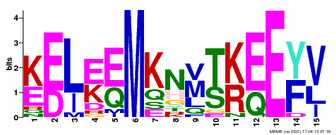 |
|  | [KE][ED][LI][EK][EQ]MKN[MV][TS][KR][EQ]E[YF][VL] |
| MOTIF-12 | 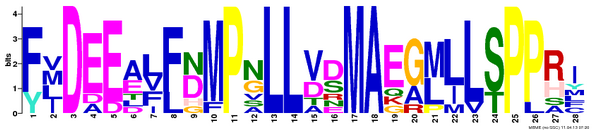 |
|  | [FY][ILMV]DEE[AE][LFV][FL][DN]MP[NG]LL[VD][DS]MAE[GA][ML][LI]L[ST]PP[RH]I |
| MOTIF-13 | 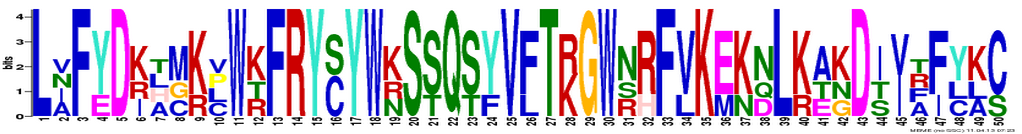 |
|  | L[AINV]F[YE]D[KIR][AHLT][MCG][KR][CLPV]W[KRT]FRY[CS]YW[KNR]S[ST]Q[ST][YF]V[FL]T[KR]GW[NRS][RH]F[VL]K[EM][KN][NDQ]L[KR][AET][KGN]D[IST][IV][AFRT][FI][YCL][KAL][CS] |
| MOTIF-14 | 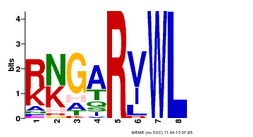 |
|  | [RK][NK]G[AT]R[VI]WL |
| MOTIF-15 | 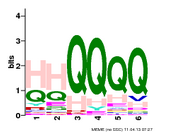 |
|  | [HQ][HQ]QQQQ |
| MOTIF-16 | 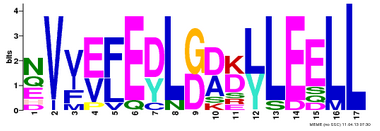 |
|  | [NEQ]V[VFI][EV][FL]E[DY]L[GD][DA][DK][LY]LEELL |
| MOTIF-17 | 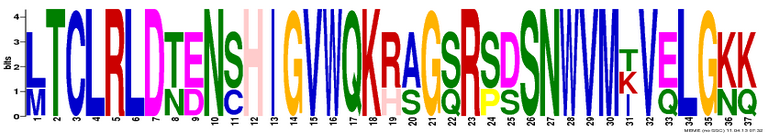 |
|  | [LM]TCLRLD[TN][ED]N[SC]HIGVWQK[RH][AS]G[SQ]R[SP][DS]SNWVM[IKT]V[EQ]LG[KN][KQ] |
| MOTIF-18 | 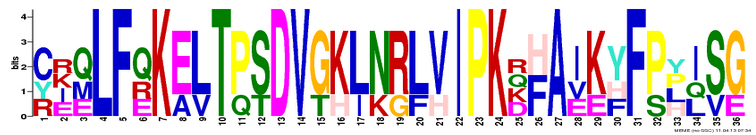 |
|  | [CRY][EIKR][QEM]LF[QER]K[EA][LV]T[PQ][ST]DV[GT][KH][LI][NK][RG][LF][VH]IPK[DKQR][FH]A[IEV][KE][YFH]F[PS][HLPY][ILQ][SV][GE] |
| MOTIF-19 | 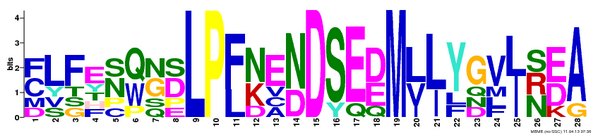 |
|  | [CF]LFE[NS][QW][GN][DS]LP[FL][NK]E[ND]DSE[DE]M[LV][LI]YGV[IL][NRS][ED]A |
| MOTIF-20 | 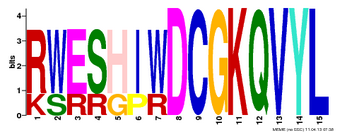 |
|  | [RK][WS][ER][SR][HG][IP][WR]DCGKQVYL |
| MOTIF-21 | 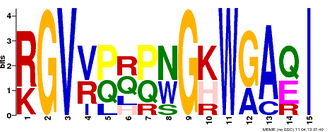 |
|  | [RK]GV[VR][PQ][QR][PQ][NW]G[KH]W[GA]A[QE]I |
| MOTIF-22 | 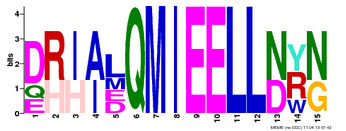 |
|  | [DEQ][RH][IH][AI][LDEM]QMIEELL[ND][RYW][NG] |
| MOTIF-23 | 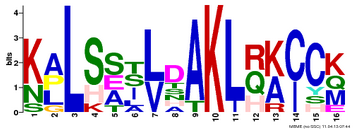 |
|  | [KN][ALP]L[SH][SAE][IST][LV]DAKL[QRH][KA][CI]C[KMQ] |
| MOTIF-24 | 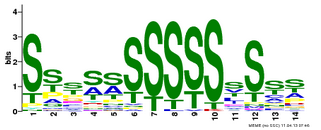 |
|  | SSSS[SA]SSS[ST]SSSSS |
| MOTIF-25 | 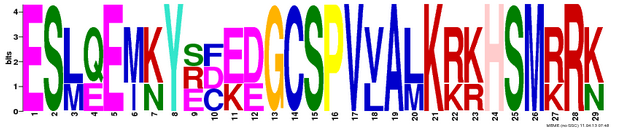 |
|  | ES[LM][QE]E[MI][KN]Y[ERS][CDF][EK][DE]GCSPV[VL]A[LM]K[RK][KR]HSM[RK]R[KN] |
