# Supplementary material for: Genome-wide survey and expression profiles of the AP2/ERF family in castor bean (Ricinus communis L.)
Source: BMC Genomics. 2013 Nov 13;14(1):785. doi: 10.1186/1471-2164-14-785 (PMC4046667; doi:10.1186/1471-2164-14-785)

Additional file 9 Sequencing quality and saturation analysis of the five libraries of root, leaf, seed 1, seed 2 and endosperm.


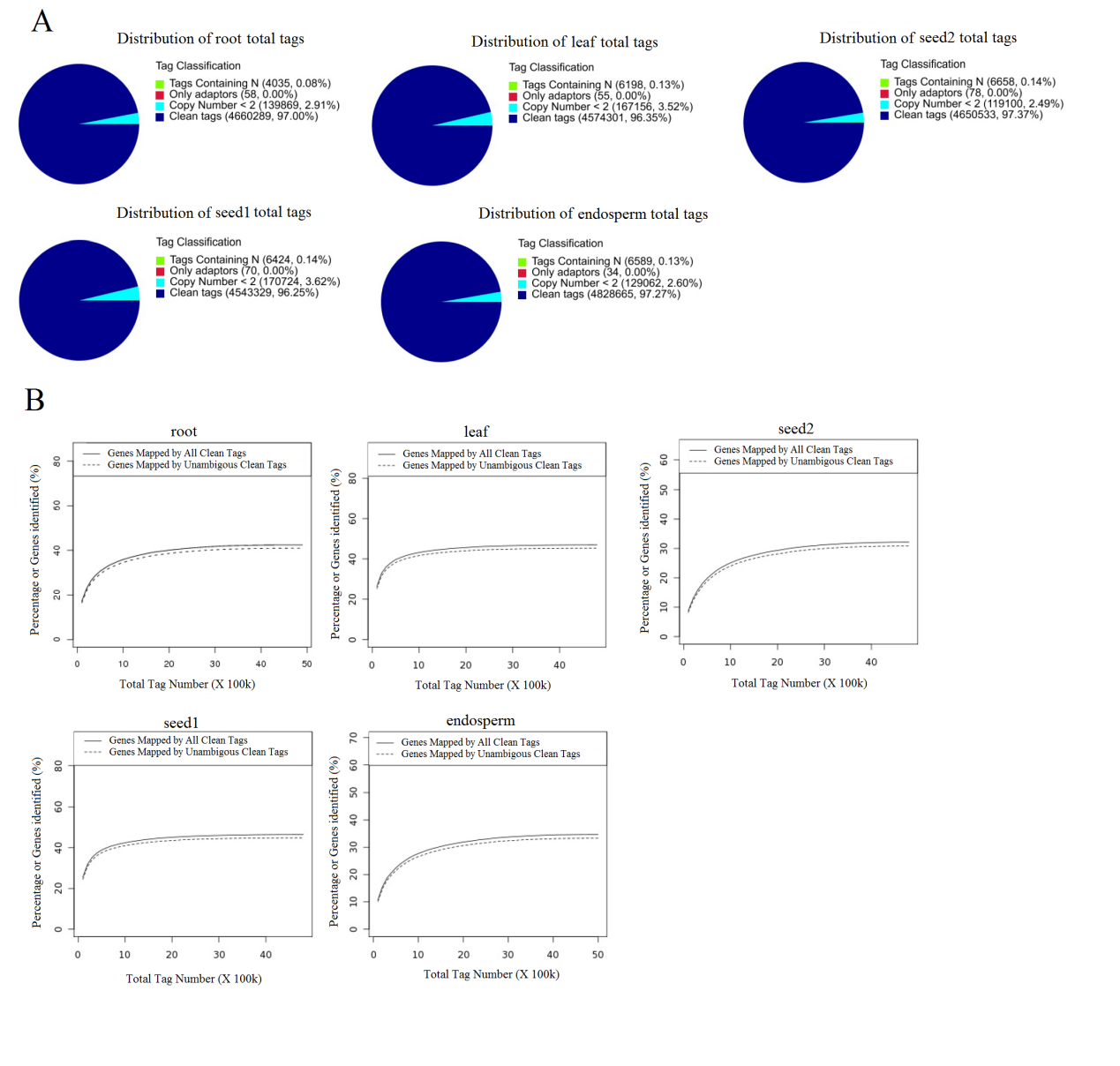

Supplement: Supplementary file 9 — Additional file 9: Sequencing quality and saturation analysis of the five libraries of root, leaf, seed 1, seed 2 and endosperm. (DOCX 429 KB) [file 12864_2013_5510_MOESM9_ESM.docx]
