# Supplementary material for: Genome-wide survey and expression profiles of the AP2/ERF family in castor bean (Ricinus communis L.)
Source: BMC Genomics. 2013 Nov 13;14(1):785. doi: 10.1186/1471-2164-14-785 (PMC4046667; doi:10.1186/1471-2164-14-785)
Supplement: Supplementary file 12 — Additional file 12: Expressional difference of AP/ERF genes between the seed 1 and the seed 2 tissues in castor bean. (DOCX 14 KB) [file 12864_2013_5510_MOESM12_ESM.docx]

Additional file 12. Expressional difference of AP/ERF genes between the seed 1 and the seed 2 tissues in castor bean

| Gene ID | Family-Group | Seed1 | Seed2 | Fold change (log2) | up-dowm |
| --- | --- | --- | --- | --- | --- |
| 29908.m006005 | A6 | 21.79 | 1.08 | -4.33 | down |
| 30190.m011323 | B1 | 7.04 | 0.43 | -4.03 | down |
| 29983.m003147 | B1 | 15.63 | 0 | -10.61 | down |
| 30190.m011321 | B1 | 18.05 | 0 | -10.82 | down |
| 27904.m000217 | B1 | 28.39 | 2.37 | -3.58 | down |
| 29640.m000403 | B1 | 51.06 | 2.15 | -4.57 | down |
| 27585.m000144 | B2 | 74.83 | 1.51 | -5.63 | down |
| 27524.m000298 | B2 | 10.34 | 0 | -10.01 | down |
| 29726.m004094 | B2 | 2.64 | 44.08 | 4.06 | up |
| 29900.m001561 | B3 | 4.62 | 0 | -8.85 | down |
| 29900.m001559 | B3 | 4.84 | 0 | -8.92 | down |
| 29895.m000321 | B3 | 9.46 | 0 | -9.89 | down |
| 30147.m014329 | B3 | 11.23 | 0 | -10.13 | down |
| 28192.m000255 | B3 | 52.16 | 5.38 | -3.28 | down |
| 30128.m008556 | B4 | 8.14 | 0.86 | -3.24 | down |
| 29863.m001066 | B4 | 31.47 | 0.43 | -6.19 | down |
| 28641.m000082 | B5 | 16.51 | 0 | -10.69 | down |
| 28752.m000339 | AP2 | 16.73 | 4.09 | -2.03 | down |
| 29169.m000017 | AP2 | 35.22 | 3.44 | -3.36 | down |
| 30069.m000440 | AP2 | 0 | 39.78 | 11.96 | up |
